# Male Central Precocious Puberty: Serum Profile of Anti-Müllerian Hormone and Inhibin B before, during, and after Treatment with GnRH Analogue

**DOI:** 10.1155/2013/823064

**Published:** 2013-11-12

**Authors:** Romina P. Grinspon, Luz Andreone, Patricia Bedecarrás, María Gabriela Ropelato, Rodolfo A. Rey, Stella M. Campo, Ignacio Bergadá

**Affiliations:** Centro de Investigaciones Endocrinológicas “Dr. César Bergadá” (CEDIE), CONICET-FEI-División de Endocrinología, Hospital de Niños Ricardo Gutiérrez, Gallo 1330, C1425EFD Buenos Aires, Argentina

## Abstract

We aimed to describe the functional changes of Sertoli cells, based on the measurement of serum anti-Müllerian hormone (AMH) and inhibin B during treatment with GnRHa and after its withdrawal in boys with central precocious puberty. Six boys aged 0.8 to 5.5 yr were included. AMH was low at diagnosis in patients >1 yr but within the normal range in younger patients. AMH increased to normal prepubertal levels during treatment. After GnRHa withdrawal, AMH declined concomitantly with the rise in serum testosterone. At diagnosis, inhibin B was elevated and decreased throughout therapy, remaining in the upper normal prepubertal range. In patients with testicular volume above 4 mL AMH remained higher in spite of suppressed FSH. After treatment withdrawal, inhibin B rose towards normal pubertal levels. In conclusion, AMH did not decrease in patients <1 yr reflecting the lack of androgen receptor expression in Sertoli cells in early infancy. Serum inhibin B might result from the contribution of two sources: the mass of Sertoli cells and the stimulation exerted by FSH. Sertoli cell markers might provide additional tools for the diagnosis and treatment followup of boys with central precocious puberty.

## 1. Introduction

Precocious puberty in boys is defined as the appearance of the initial signs of sexual maturation at an age that is more than 2–2.5 SD below the population mean, which is 9 yr for boys [1]. Precocious puberty can be gonadotropin dependent due to the early activation of pulsatile gonadotropin secretion or independent of gonadotropins, as is the case with testotoxicosis or Leydig cell tumours [2]. The aetiology of an early activation of pulsatile gonadotropin secretion may be secondary to an organic lesion within the central nervous system (hydrocephaly, tumours, meningitis, etc.) or due to activating mutations of the genes encoding kisspeptin [3] or its receptor GPR54 [4]. However, in many cases the cause that triggers the onset of puberty cannot be identified. Gonadotropin-dependent precocious puberty is more prevalent in girls; nevertheless, the proportion of cases of precocious puberty secondary to organic lesions is higher in boys [2].

Gonadotropin-releasing hormone analogues (GnRHa) is the treatment of choice for gonadotropin-dependent precocious puberty of central origin. Therapy induces a reversible pituitary desensitization of GnRH receptors, thus suppressing the production of gonadotropins and subsequently that of sex steroids. This slows pubertal progression and decreases the rate of linear growth and skeletal maturation. Slowing the advancement of bone age is the primary goal of GnRHa therapy in order to improve final height [1, 2].

Monitoring treatment effectiveness during GnRHa therapy resides in multiple items. Growth velocity, secondary sexual characteristics, and bone maturation are the main clinical parameters. Serum LH and FSH measured by ultrasensitive assays are used for the assessment of pituitary desensitization [5, 6], and serum testosterone levels are used to monitor testicular response to treatment.

Measurement of testicular volume is clinically utilized for the assessment of pubertal development in boys [7]; the increase in testicular volume beyond 4 mL is typically used to define the onset of puberty, either normal or precocious. The initial increase in testicular volume is due to Sertoli cell proliferation induced by FSH. Subsequently, testosterone provokes Sertoli cell maturation—characterized by Sertoli cell proliferation arrest—and triggers pubertal spermatogenesis in the seminiferous tubules, which mainly accounts for the dramatic enlargement of testis size to 15–25 mL [8]. Other early signs of pubertal Sertoli cell maturation, which occur even before serum testosterone rises, are the changes in serum levels of two specific Sertoli cell products: the decrease in anti-Müllerian hormone (AMH) [9–11] and the increase in inhibin B [12–14].

In the present study, our primary objective was to assess functional changes in Sertoli cells, based on the measurement of serum AMH and inhibin B during treatment with GnRHa and after its withdrawal in boys with central precocious puberty.

## 2. Methods

### 2.1. Patients

Boys diagnosed with central precocious puberty at the Division of Endocrinology of the Hospital de Niños Ricardo Gutiérrez, a tertiary paediatric public hospital in the city of Buenos Aires, Argentina, and treated with GnRHa between 1995 and 2013 were included in the study. Patients were excluded if history chart or results of serum hormone determinations (FSH, LH, testosterone, and AMH) were not complete or available. A clinical examination was performed at each visit to assess pubertal stage according to Marshall and Tanner [7] and to determine testicular volume by comparison with Prader's orchidometer [15]. The mean of the volume of both testes was reported. LH, FSH, testosterone, inhibin B, and AMH serum concentrations were assessed periodically before, throughout, and after treatment with GnRHa. The study was approved by the institutional review board. The need for informed consent was waivered owing to the observational design of the study, in which most of the procedures followed the standard care of patients with central precocious puberty, and because results of serum AMH and inhibin B levels were not considered for decision making.

### 2.2. Hormone Assays

AMH was determined using an ultrasensitive enzyme linked immunoassay specific for human AMH (Immunotech, Beckman-Coulter Co., Marseilles, France), following the manufacturer's instructions as previously published [16]. The analytical sensitivity of the assay, defined as the lowest AMH concentration significantly different from the calibrator zero, was 2.3 pmol/L. Intra- and interassay coefficients of variation were, respectively, 10.5% and 9.4% for a serum AMH concentration of 700 pmol/L and 11.1% and 12.8% for a serum AMH concentration of 7 pmol/L. Reference values were taken from our own data [16].

Serum inhibin B was measured using two-site enzyme-linked immunosorbent assays (Oxford Bio-Innovation Ltd, Oxon, UK) as previously described. Recombinant inhibin B (Genentech, San Francisco, CA, USA) was used as standard. The assay sensitivity was 15 pg/mL. Intra- and interassay coefficients of variation were below 10% for all assays. Reference values were taken from our own data [12]. 

Serum FSH and LH were measured by time-resolved immunofluorometric assays (IFMA, DELFIA; PerkinElmer, Inc. by Wallac Oy, Turku, Finland). The functional sensitivities were 0.05 and 0.10 IU/L, according to the 2nd WHO IS 80/552 for LH and IRP 94/632 for FSH, respectively. Intra- and interassay coefficients of variation for IFMA method were ≤3.2 and ≤7.3% for LH and ≤2.3 and ≤5.2% for FSH, as previously published [17]. 

### 2.3. Statistics

Hormone concentrations below the limit of the assay detection were assigned a concentration equivalent to the minimum detectable value in the respective assay. Data are expressed as median and range. Multiple regression analysis was performed to assess the association between serum inhibin B levels and testis volume during followup after GnRHa treatment. The level of significance was set at *P* < 0.05. Statistical analyses were performed using GraphPad Prism version 5.01 for Windows (GraphPad Software, San Diego, CA, USA) and IBM SPSS Statistics (IBM Corporation, Somers, NY, USA).

## 3. Results

Six boys with central precocious puberty were included in the study (Table 1). Their median age at diagnosis was 3 years (range 0.8–5.5) with a bone age of 8.5 years (range 1.25–10.5). In three cases, precocious puberty was due to a hypothalamic hamartoma and in the other three it was idiopathic. Patients were treated with triptorelin acetate every 28 days at a 110–190 *μ*g/kg dose. GnRHa treatment was stopped at a median age of 10.1 (range 8.9–10.7). Patient 5 was still in treatment at the moment of the study.

**Figure 1:**
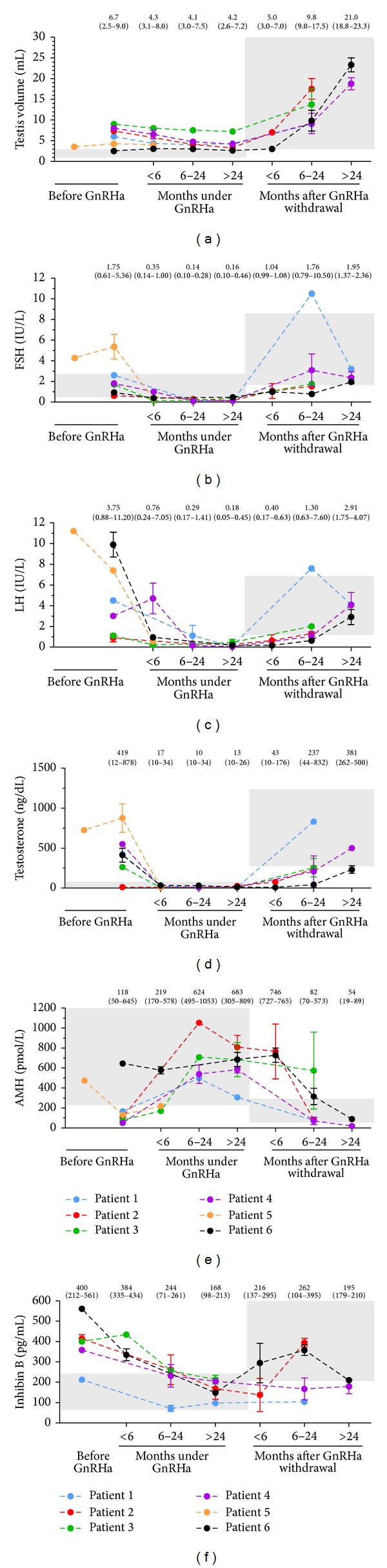
Testicular volume and hormone levels of boys with central precocious puberty before, during, and after withdrawal of GnRH analogue treatment. Medians and ranges are shown on the top of each figure for each time point.

At diagnosis, testicular volume was between 2 and 9 mL (Figure 1(a)), FSH was 1.75 IU/L (range 0.61–5.36) (Figure 1(b)), median basal LH was 3.75 IU/L (range 0.88–11.20) (Figure 1(c)), and testosterone was 419 ng/dL (range 12–878) (Figure 1(d)). Median peak LH after an acute GnRH test was 35.30 IU/L (range 6.90–84.90) (Table 1). Serum AMH was low in the four patients older than 1 year (median 67 pmol/L, range 50–165) but was within the normal range in the two patients aged ≤1 year (645 and 475 pmol/L) (Figure 1(e)). Interestingly, in Patient 5, who was diagnosed at 12 months of age, AMH (475 pmol/L) was within the normal prepubertal range. Due to social difficulties GnRHa treatment could not be started until the age of 2.2 yr when serum AMH had decreased to 131 pmol/L. Before treatment serum inhibin B was elevated (400 pg/mL, range 212–560) (Figure 1(f)).

Throughout treatment with GnRHa, testis volume decreased but remained moderately above the normal prepubertal size in most patients (Figure 1(a)). As expected, serum gonadotropins and testosterone showed a progressive decrement toward normal prepubertal levels and remained so until GnRHa withdrawal, when they increased again to pubertal levels (Figures 1(b)–1(d)). AMH increased progressively and remained within the normal prepubertal range during the whole treatment (Figure 1(e)). After GnRHa withdrawal, AMH progressively declined concomitantly with the rise in serum testosterone, as in normal puberty [16, 18].

**Figure 2:**
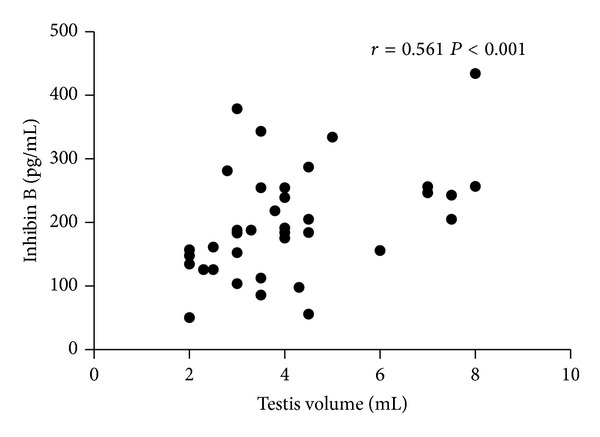
Correlation between testicular volume and serum inhibin B levels in boys with central precocious puberty during GnRH analogue treatment.

Serum inhibin B levels decreased moderately throughout GnRHa therapy attaining its nadir after 24 months of treatment. Thereafter, they remained in the upper half of normal prepubertal age range (Figure 1(f)). A multiple regression analysis using patient and testis volume as the independent variables and inhibin B levels as the dependent variable showed a significant positive correlation between testicular volume and serum inhibin B levels during treatment (*r* = 0.561  *P* < 0.001) (Figure 2). Interestingly, during GnRHa treatment the lowest levels of inhibin B were observed in Patients 1, 2, and 6, in whom testicular volume achieved normal prepubertal range, whereas inhibin B remained higher in Patients 3 and 4, who maintained testicular volume above 4 mL in spite of suppressed FSH levels. After treatment withdrawal, inhibin B rose toward normal pubertal levels concomitantly with the reactivation of the gonadotropin axis. 

## 4. Discussion

By means of serum AMH and inhibin B assessment, this work depicts the functional changes occurring in the seminiferous tubule compartment—and especially in Sertoli cells—in response to GnRHa treatment in boys with central precocious puberty. Our results show that the abnormally early maturation process suffered by Sertoli cells in boys with precocious puberty in response to testosterone is reversible when patients are efficaciously treated with GnRHa, most probably reflecting the decrease to prepubertal levels in intratesticular testosterone concentration. On the contrary, inhibin B levels do not always decrease to prepubertal values although FSH secretion is also curtailed by GnRH administration, most probably indicating that the increased mass of Sertoli cells is not fully reverted and continues secreting inhibin B independently of FSH stimulation.

Most of our patients had low serum AMH levels for their chronological age when central precocious puberty was diagnosed, showing the well-known inhibition exerted by androgens on Sertoli cell AMH production. The inhibitory action of androgens predominates over the stimulatory effect of FSH on Sertoli cell AMH expression [19, 20]. Conversely, FSH action on Sertoli cells is reflected by the initial increase in testicular volume and serum inhibin B. AMH downregulation and inhibin B upregulation also reflect the entry into meiosis of germ cells. In fact, the onset of meiosis is associated with a further decline in AMH expression, independently of testosterone, during pubertal development [21]. Concerning inhibin B production by the testis, the immature Sertoli cell synthesizes the *α*/*β*B dimer, whereas the pubertal Sertoli cell essentially produces the *α* subunit under FSH control; the *β*B subunit expression is mainly regulated by local factors produced by meiotic germ cells [22]. 

During GnRHa treatment AMH attained normal prepubertal levels, as previously described [9], which accounts for the usefulness of Sertoli cell serum markers to monitor the efficacy of GnRHa treatment in order to produce a sufficient decrease in gonadotropin production so as to lower intratesticular androgen action to prepubertal levels. The functional maturation of Sertoli cells induced at pubertal onset, reflected in AMH downregulation by androgens, seems to be a completely reversible process when treated soon before its establishment, as was the case in Patients 1–4 of our series and in previously published cases [9]. On the contrary, Sertoli cells do not revert to a fully immature state if the action of intratesticular androgens has persisted for years or decades, as shown in adult males receiving GnRHa treatment for prostate cancer [23].

A noteworthy observation in our study was that serum AMH remained in the normal prepubertal range in patients with central precocious puberty diagnosed within the first year of life, suggesting that the elevated androgen levels were unable to inhibit AMH production in young infants. Interestingly, testicular volume also remained within the prepubertal range in these patients, probably indicating that pubertal spermatogenesis had not been triggered. These observations are in line with the recent description that Sertoli cells are physiologically insensitive to the direct action of androgens in the first year after birth because they do not express the androgen receptor until later in life [24–26]. Concordantly, in our Patient 5, AMH was at prepubertal levels when diagnosed at 12 months of age and, because treatment could not be immediately installed, serum AMH decreased to pubertal levels at age of 2.2 years, supporting the concept of a gradual increase in androgen receptor expression within the Sertoli cell population.

At diagnosis, serum inhibin B levels were elevated in all patients, resembling what occurs under normal pubertal development at Tanner stages II to III. Treatment with GnRHa resulted in a reduction in serum inhibin B levels in all patients. However, the magnitude of this decrement was variable amongst patients. In some patients, inhibin B remained in the upper normal range during treatment, in correlation with the persistence of moderately elevated testis volume, despite suppressed FSH secretion. This probably reflects that the increased mass of Sertoli cells induced by elevated FSH until diagnosis is not fully restored to the prepubertal stage and continues secreting inhibin B, independently of FSH stimulation [14]. We believe that serum levels of inhibin B reflects the addition of two pools: a basal one related to the mass effect of the Sertoli cell population independent of the gonadotropins and a second one related to the stimulation exerted by FSH. The mass effect is evidenced by the observation that testicular volume remains over the normal prepubertal volume in a subset of patients under GnRHa treatment, concomitantly with higher serum inhibin B levels. It could be argued that these elevated serum inhibin B levels result from an insufficient inhibition of the gonadotropin axis with GnRHa treatment. This seems unlikely, however, since serum levels of LH and testosterone were efficaciously suppressed and resulted in a normalization of serum AMH.

In summary, we describe the different functional changes observed in Sertoli cells of boys with central precocious puberty according to their age at the moment of diagnosis. Sertoli cell markers provide additional tools to diagnose central precocious puberty in boys older than 1 year and to monitor GnRHa treatment efficacy. The observation that serum AMH does not decrease in patients below 1 year confirms the lack of androgen receptor expression in Sertoli cells in early infancy. Finally, we hypothesize that serum levels of inhibin B represent the addition of two pools, one related to the stimulation exerted by gonadotropins and another one to the mass effect of the Sertoli cell population independent of the gonadotropins.

## Figures and Tables

**Table 1:** Characteristics of boys with central precocious puberty included in this study.

	Age at diagnosis (years)	Bone age at diagnosis (years)	Peak LH at diagnosis (U/L)	Peak FSH at diagnosis (U/L)	Etiology	Age at GnRHa start (years)	Age at GnRHa end (years)
Patient 1	5.5	8.5	35.30	2.10	Idiopathic	5.6	8.9
Patient 2	4.3	8.5	6.90	0.70	Hypothalamic hamartoma	4.8	10.3
Patient 3	4.3	10.5	13.40	3.10	Hypothalamic hamartoma	4.3	9.8
Patient 4	1.7	3.6	37.00	2.30	Hypothalamic hamartoma	2.0	10.7
Patient 5	1.0	1.3	32.90	7.40	Idiopathic	2.2	N.A
Patient 6	0.8	2.0	84.90	1.70	Idiopathic	1.1	10.0

N.A: not applicable.
